# Five studies evaluating the impact on mental health and mood of recalling, reading, and discussing fiction

**DOI:** 10.1371/journal.pone.0266323

**Published:** 2022-04-08

**Authors:** James Carney, Cole Robertson

**Affiliations:** 1 The London Interdisciplinary School, London, United Kingdom; 2 Center for Language Studies, Radboud University, Radboud, Netherlands; University of St Andrews, UNITED KINGDOM

## Abstract

Does reading fiction improve mental health and well-being? We present the results of five studies that evaluated the impact of five forms of exposure to fiction. These included the effects of recalling reading fiction, of being prescribed fiction, of discussing fiction relative to non-fiction, and of discussing literary fiction relative to best-seller fiction. The first three studies directly recruited participants; the final two relied on scraped social media data from Reddit and Twitter. Results show that fiction can have a positive impact on measures of mood and emotion, but that a process of mnemonic or cognitive consolidation is required first: exposure to fiction does not, on its own, have an immediate impact on well-being.

## Introduction

The claim that exposure to literature can have a positive impact on mental well-being has been visible for some time now. ‘Creative bibliotherapy’, as this view has come to be known, argues that the cognitive effects engendered by poetry, fiction, and drama may be of value in treating mental health conditions [1–6]. In one formulation, ‘attentive immersion in great literature can help relieve, restore, and reinvigorate the troubled mind—and can play a part in relieving stress and anxiety, as well as other troubled states of mind’ [7]. In a world where the need for affordable mental health resources outstrips the ability to supply them, this is an attractive proposition. It is unsurprising, therefore, that numerous popular authors have been enthusiastic in their endorsement of reading literature as a therapeutic intervention [8–14], or that services such as ReLit, The Reading Agency, and The School of Life seek to pair troubled readers with literature that will ostensibly improve their state of mind.

But enthusiasm for an intervention is not an argument in its favour. If anything, such enthusiasm mandates that one should even more circumspect than usual in the assessment of the evidence in support of the intervention. ‘The first principle’, as Richard Feynman observed, ‘is that you must not fool yourself—and you are the easiest person to fool’ [15]. A programme like creative bibliotherapy, which promises improvements to mental health by way of a high-prestige, hedonic activity like reading literature, needs a solid evidence base if we are to guard against its subjective attractiveness. As the evidence revolution in medicine has made clear, arguments from experience and expert-led judgment—however well-intentioned they may be—come a poor second to statistical rigour and data-driven conclusions [16]. If creative bibliotherapy is to be credited with genuine therapeutic efficacy, therefore, it needs to be tested using the full resources of experimental evaluation.

This is especially needful given the current state of the field. Though creative bibliotherapy has been sporadically subjected to empirical assessment, the results do not yield a consistent picture and hypotheses have, in general, not been well formulated (see Troscianko [17] for the best appraisal of the literature). To start with the issue of evidence, it is certainly true that a reasonable number of studies attribute therapeutic change to the experience of reading literature. For example, group-based studies record positive impact on measures of cognitive performance like concentration, memory, social skills, and creativity, as well as affective change in the direction of increased confidence, fewer feelings of isolation, and improvement on markers of depression [6, 18–20]. Paralleling this, studies focused on individual reading argue for positive impacts on adolescent aggression [21, 22], expressiveness in ill children [23], self-confidence and functioning [5], interpersonal empathy [24, 25], and theory of mind [24] However, results do not seem to generalise, with a meta-analysis by Montgomery and Maunders [26] finding only minor evidence for the efficacy of creative bibliotherapy in addressing PTSD, and Glavin and Montgomery [27] finding no therapeutic effects at all. Perhaps most worryingly, Troscianko [17] presents evidence that readers with eating disorders recall having their symptoms exacerbated by fiction that thematises the experience of having an eating disorder. If so, creative bibliotherapy violates the very first injunction of therapeutic intervention—to do no harm.

But the problem is not solely evidential; difficulties also emerge at the conceptual level. Most obviously, terms like ‘literature’ and ‘mental health’ need to be clearly defined. It is not at all clear, for instance, that there is any common feature underlying the heterogenous collection of works, genres and individual styles that comprise what is generally accepted as literature. Even if this were not the case, it would remain unlikely that literature would impact in the same way upon the 297 or so disorders that the DSM 5 defines as constituting the repertoire of mental illness [28]. Finally, even conceding that literature (by whatever definition) has a positive impact on mental health, there remains an explanatory gap concerning what causal mechanisms may meditate or moderate this impact. So far as it has been theorised at all, the main approaches seem to volunteer literature as a form of cognitive behaviour therapy (CBT), such that literary texts allow readers to re-frame their orientation to the world by allowing the self to be refracted through identification with a third-party perspective [29, 30]. Though such ideas have an intuitive appeal, the fact is that they often better resemble the hard-to-test constructions of psychoanalysis than they do the pragmatic paradigm of CBT. While this does not make them wrong, it does foreclose the possibility of other (possibly more mundane) causal mechanisms being identified.

These considerations, in combination with the intellectual importance of establishing the relation between culture and mental health, are what motivated us to assess the therapeutic value of literature by way of the five studies presented here. While no authors can claim to exhaust a topic, our goal was to conduct as systematic an evaluation as possible of how one form of literature—fiction—impacts on mental health, while allowing for the different modes of encounter that characterise exposure to fiction. We chose fiction for the simple reason that most people experience literature through fiction, so any results would have the widest applicability. This is not to deny that fiction can attracts problems of definition in the same way as literature [31, 32], but for our purposes we take fiction to be the linguistically mediated evocation of a counterfactual reality in narrative form. The principal mental health conditions we evaluated were depression and anxiety—once more for the simple reason that these conditions, whether individually or in co-morbid form, are the most common mental health presentations [33]. Usefully, however, both conditions are also associated with psychometrically validated tests that allow for them be easily measured. With that said, two of the studies did not use such tests due to the fact that their data was scraped from social media, and relied instead on linguistic measures of mental state using the valence-arousal-dominance model of emotion [34] and an absolute words measure of mental distress [35].

Where our main innovation lay was with respect to the mode of encounter with fiction. Fiction can be experienced in many different contexts, and it is at least *prima facie* plausible that any cognitive or emotional impact it might have will be shaped by the form of this experience. For instance, being required to read a novel as part of one’s high school education will result in a very different experience from freely choosing to read that novel for personal pleasure. Any thorough appraisal of creative bibliotherapy should therefore take some account of this variation. We did this by identifying five different modes of encounter with fiction and implementing an experimental design that reflected each mode.

1. *Recalled impact of reading fiction*: The fiction we are reading right now is always dwarfed by the amount fiction we have previously read. Moreover, the impact of fiction may involve periods of reflective consolidation that last months or even years [36]. This means that there are good grounds for evaluating fiction with respect to its recalled effects, as well as its immediate effects.
2. *Impact of prescribed fiction*: Most people first encounter literary fiction in a classroom context, where texts are prescribed for mandatory reading from a canon of classics. The assumption here seems to be that forced exposure will have an improving effect, though this is by no means empirically established. Any assessment of creative bibliotherapy should therefore investigate the effects of prescribed fiction.
3. *Impact of chosen fiction*: Habitual readers of fiction—i.e. those who gain most hedonic value from reading—will have well-developed tastes that guide what they choose to read. If fiction has a therapeutic effect, then it is to be expected that the act of choosing a text will impact on its effectiveness. This gives a third modality of encounter that needs to be contrasted with prescribed fiction.
4. *Impact of discussing fiction*: Reading is typically seen as a solitary exercise, but it is also particularly effective at stimulating collective discussion. This happens at a professional level in the form of cultural journalism and academic criticism, but it is no less present in informal reading groups and online forums. As in-person reading groups are explored by one of us in another study [37], we here look exclusively at the effects of discussing reading in an online context.
5. *Impact of fiction quality*: Judgments of artistic quality are notoriously subjective, but most readers are prepared to distinguish between fiction as entertainment and fiction as an aesthetic phenomenon. As exposure to different types of fiction may have an impact on therapeutic effects, any thorough evaluation needs to allow for variation in fiction quality.

As not all of these forms of encounter with fiction could be assessed using the same methods, our first three studies were performed by directly recruiting online participants using Amazon’s Mechanical Turk platform, while the last two relied on social media data from Twitter and Reddit. The Reddit data was accessed through the Reddit API; the Twitter data was obtained using data scraping methods. As all usernames were cryptographically hashed at point of acquisition and no text data published, we cannot make inferences about the mental states of individual users and are therefore compliant with the terms of use of both platforms. Inevitably, presenting methodologically distinct studies together in this way means there must be some compression of information. Our view is that this compression is a price worth paying, given that the creative bibliotherapy research is widely dispersed across the academic literature on account of not obviously belonging to either the humanities or the experimental social sciences. By the same token, we recognise that these studies may be of interest to readers who do not have training in quantitative methods; every effort will therefore be made to keep them accessible, consistent with accurately reporting the relevant results.

All studies that involved the collection and retention of unpublished data received ethics clearance from the Brunel University London College of Business, Arts, and Social Sciences Research Ethics Committee (Ref. 7863-A-Jan/2018–10690–1). Where study participants were directly recruited, they were paid the UK living wage on a pro-rata basis.

## Study 1—Impact of recalling fiction

Our first study evaluated the hypothesis that recalling literary fiction has a therapeutic impact. The problem it poses is that different texts may not impact in the same way, due to variations between the texts; as already noted, ‘literature’ is not a well-defined phenomenon. As there is no way to entirely resolve this problem, we compromised by prompting responses around 12 well-known novels and measuring the emotional character of the language they use by way of word norm data. However, though this provided useful pilot data, our aim was not to establish the effect of these specific novels, so we also allowed participants to answer on other texts that we did not directly mention. Therapeutic change was measured using CORE-OM and PHQ scales, as well as a search for meaning measure. Given that both the word norm data and therapeutic measures feature in the subsequent studies, we deliver the background exposition for all of them in the present study.

### Materials and methods

#### Participants

Participants were recruited online using TurkPrime, an implementation of Amazon’s Mechanical Turk platform [38] on the basis of having read at least one of the twelve experimental texts. With a view to ensuring quality of response, only those participants who had at least a 97% approval rating from a minimum of 500 previous tasks were admitted. A total of 151 participants were recruited. Three participants were excluded: one who entered their sex as ‘other’ (because estimating effects of sex with only one observation in the ‘other’ category was not reliable) and two because their global distress change scores were more than 4.4 standard deviations from the mean and their inclusion in later regression analyses was exerting disproportionate influence on the estimation of effects. This left a final sample of *N* = 148 (76 females).

#### Procedure

Participants were asked to answer on two texts. The first involved participants in selecting a text from a list and estimate how long it had been since they had read it; a validity check was included by asking them to name two characters from the chosen text in a free-text field. After doing this, they were invited to recall their mental state before encountering the text, and then complete the CORE-OM inventory for the first time (note that we were less interested in the actual mental state than in their reconstruction of that that mental state might have been). Upon completion, participants were requested to rehearse the events and world of the text and immerse themselves as much as they could in the experiences they had when they were reading. After this, they once more completed the CORE-OM—though this time with reference to their recalled mental state after reading. This procedure was repeated for a second text, with an open text field—’None of the above’—provided if the participant had read only one text on the list. Participants were then required to answer the PHQ-4 inventory for depression and anxiety, complete the search for/presence of meaning question inventory, and give a Likert-scale rating of how important they felt fiction to be in their personal lives. (Note that this study centred on the effects of recalling reading the text and not actually reading the text).

A control condition was not included due to the difficulty in identifying a meaningful comparator. Neutral textual prompts—such as the Wikipedia articles on factual topics used in Study 2 and Study 3—are inherently unmemorable, and unlikely to prompt any recollection. Non-textual prompts (such as thinking about a recalled event or experience), on the other hand, cannot be guaranteed to be neutral due to variation in personal experience. As these reasons preclude the inclusion of a meaningful control, our study took the null hypothesis—that recalling fiction has no effect on well-being as our comparator.

#### Materials

Study materials consisted of twelve novel-length fictional narratives, selected from an undergraduate reading list in English literature at Brunel University London. The texts referenced were Charlotte Brontë’s *Jane Eyre* (1847), George Eliot’s *Adam Bede* (1859), Mary Shelley’s *Frankenstein* (1818), Emily Brontë’s *Wuthering Heights* (1847), Mary Elizabeth Braddon’s *Lady Audley’s Secret* (1862), Elizabeth Gaskell’s *North and South* (1855), H.G. Wells’s *The Island of Doctor Moreau* (1896), Thomas Hardy’s *Tess of the D’Urbervilles* (1891), Charles Dickens’s *Great Expectations* (1860), Joseph Conrad’s *Heart of Darkness* (1899), and Charles Dickens’s *Hard Times* (1854). In the event that participants had read only one of these texts, they were allowed to answer on a different text of their choosing.

#### Independent variables

Linguistic variation between texts was measured using word norms for valence, arousal, dominance (VAD) and concreteness (C). In this connection, a word norm is the average response to a particular word on a given dimension. Typically, word norms are the result of mega-studies, where large corpuses of words are assessed for the response that individual words evoke [39, 40]. Here, we used the VAD norms established in Warriner, Kuperman, & Brysbaert [41] and the concreteness norms published in Brysbaert, Warriner, & Kuperman [42].

The value of word norms is that they provide a low dimensional proxy for emotional variation. That is, according to dimensional models of emotion, each discrete emotion can be represented in terms of an underlying set of finite components [34, 43]. The VAD model identifies these components as valence, arousal, and dominance, where valence measures how positive (or negative) an emotion is felt to be, arousal measures how energising (or sedating) an emotion is felt to be, and dominance measures how in control (or controlled) and emotion is felt to be. Thus, anger is negatively valent, positively arousing, and negatively dominant, while happiness is positively valent, neutrally arousing and positively dominant.

Here, we used the VAD norms established in Warriner, Kuperman, & Brysbaert [41] and the concreteness norms published in Brysbaert, Warriner, & Kuperman [42]. Warriner et al. (2013) present VAD ratings for 13,914 common English words, thereby providing an empirically validated way of assessing the overall emotional impact of a word, making mean VAD easy to calculate. The concreteness norms published in Brysbaert et al. [42] provide ratings of 37,058 English words with respect to how concrete or abstract they are felt to be.

**Fig 1:**
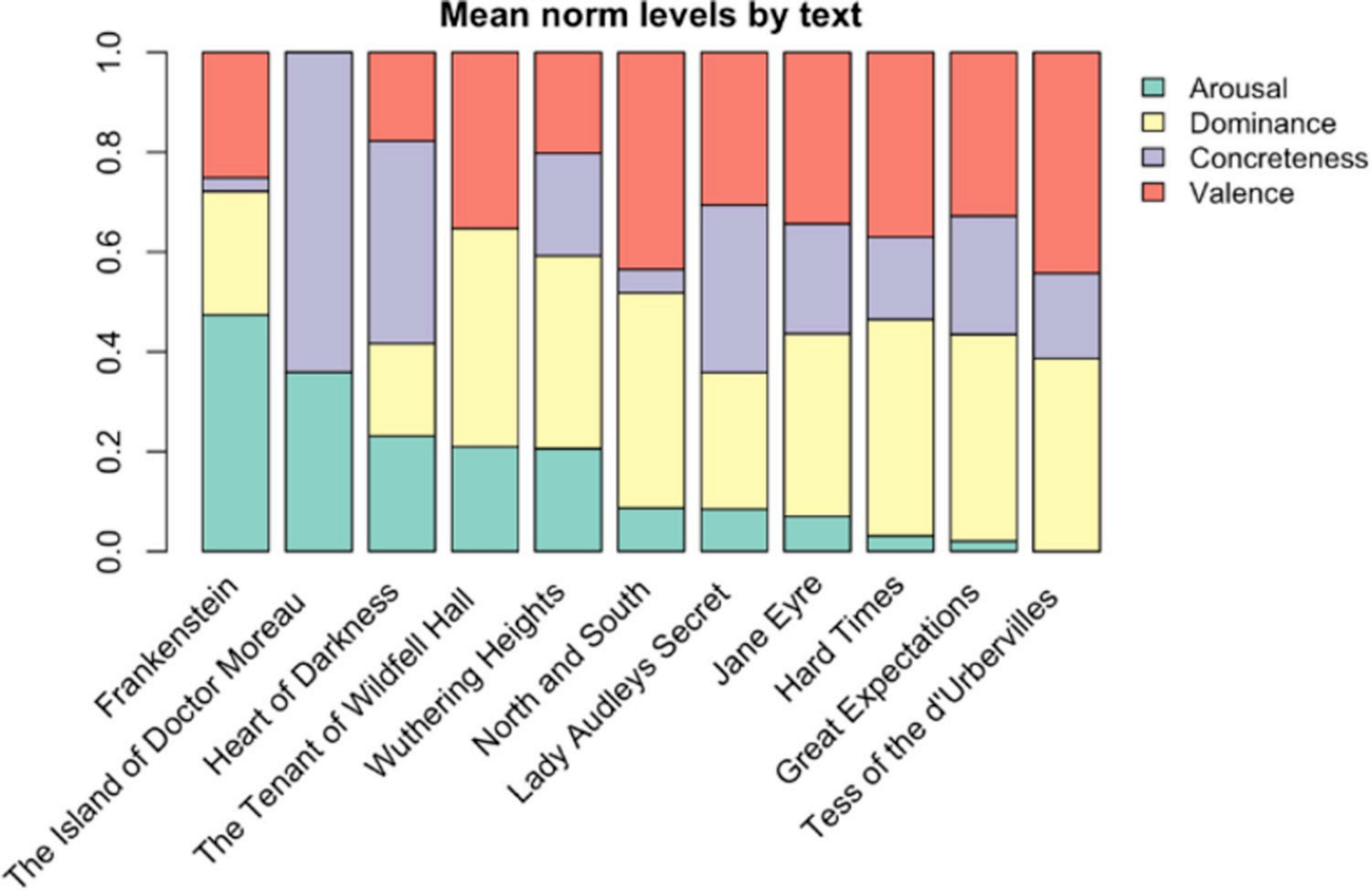
Variables are scaled between 0 and 1 such that a text with a 0 score represents the lowest proportion of that variable.

VAD+C levels were calculated by processing electronic copies of each text sourced at Project Gutenberg. In detail, this involved tokenizing each text into words, removing stop words (e.g. ‘to’, ‘from’, ‘for’, ‘the’, etc.), lemmatizing the remaining words into their root form (i.e. ‘running’, ‘ran’, ‘runs’ all reduce to ‘run’), and calculating the mean scores using values from word norm databases. This process was automated for future use by creating a custom process function built using the python spaCy natural language processing (NLP) library [44] Fig 1 shows the relative proportions of VAD+C in each text, normalized between 0 and 1, meaning that the text with the lowest value for a variable shows that variable as absent: i.e. *Tess of the d’Urbervilles* has the lowest value for arousal, while the Island of Doctor Moreau has the lowest values for valence and dominance.

#### Dependent and moderating variables

*Therapeutic impact*. Change in recalled pre- and post-reading states was measured using the Clinical Outcomes in Routine Evaluation Outcome Measure (CORE-OM)—a 34-item inventory that is typically used in the evaluation of psychotherapeutic interventions [45]. This battery of questions establishes change on four dimensions: well-being (W), problems or symptoms (P), functioning (F), and risk (R)—with the mean of these scores being used to define a compound variable called ‘global distress’. Typical questions include ‘Over the last week I have felt like crying’ (W), ‘Over the last week I have felt tense, anxious or nervous’ (P), ‘Over the last week I have felt able to cope when things go wrong’ (F), ‘Over the last week I have felt like hurting myself’ (R). Small changes were made in the wording of the CORE-OM to make it consistent with being applied to the pre- and post-reading state. The change in score between one completion of the survey and another can be taken as a measure of therapeutic impact. As a positive change score would have indicated an increase in distress, change scores were reverse coded such that positive scores always denote an improvement in symptoms.

*Depression and anxiety*. As a key item of inquiry concerns how literary materials might differentially impact on mental health conditions, interpersonal variation with respect to the two most common conditions—anxiety and depression—was recorded. This was done using the PHQ-4 screening scale for anxiety and depression [46]. The objective of this scale is to establish underlying propensity towards anxiety or depression (or both) for screening purposes. Participants are asked to score how much over the last two weeks they have been bothered by problems like ‘Not being able to stop or control worrying’ (anxiety) or having ‘Little pleasure or interest in doing things’ (depression).

*Search for/presence of meaning*. In a previous study, we showed that the tendency to search for meaning in one’s life moderated responsiveness to a literary text—with individuals scoring highly being more receptive to the text [47]. This suggests that search for meaning may play an important role in susceptibility to any therapeutic impacts that literary texts may have. Search for/presence of meaning was measured using the questionnaire developed in Steger, Frazier, Oishi, & Kaler [48]. This asks respondents to rate questions like ‘I am always looking to find my life’s purpose’ or ‘I understand my life’s meaning’, with a view to establishing whether they experience meaning as absent or present in their lives (and care about the fact). It outputs two scores: ‘search for meaning’ and ‘presence of meaning’, with there being a typically inverse relation between the two.

### Results

#### Main hypothesis

**Fig 2:**
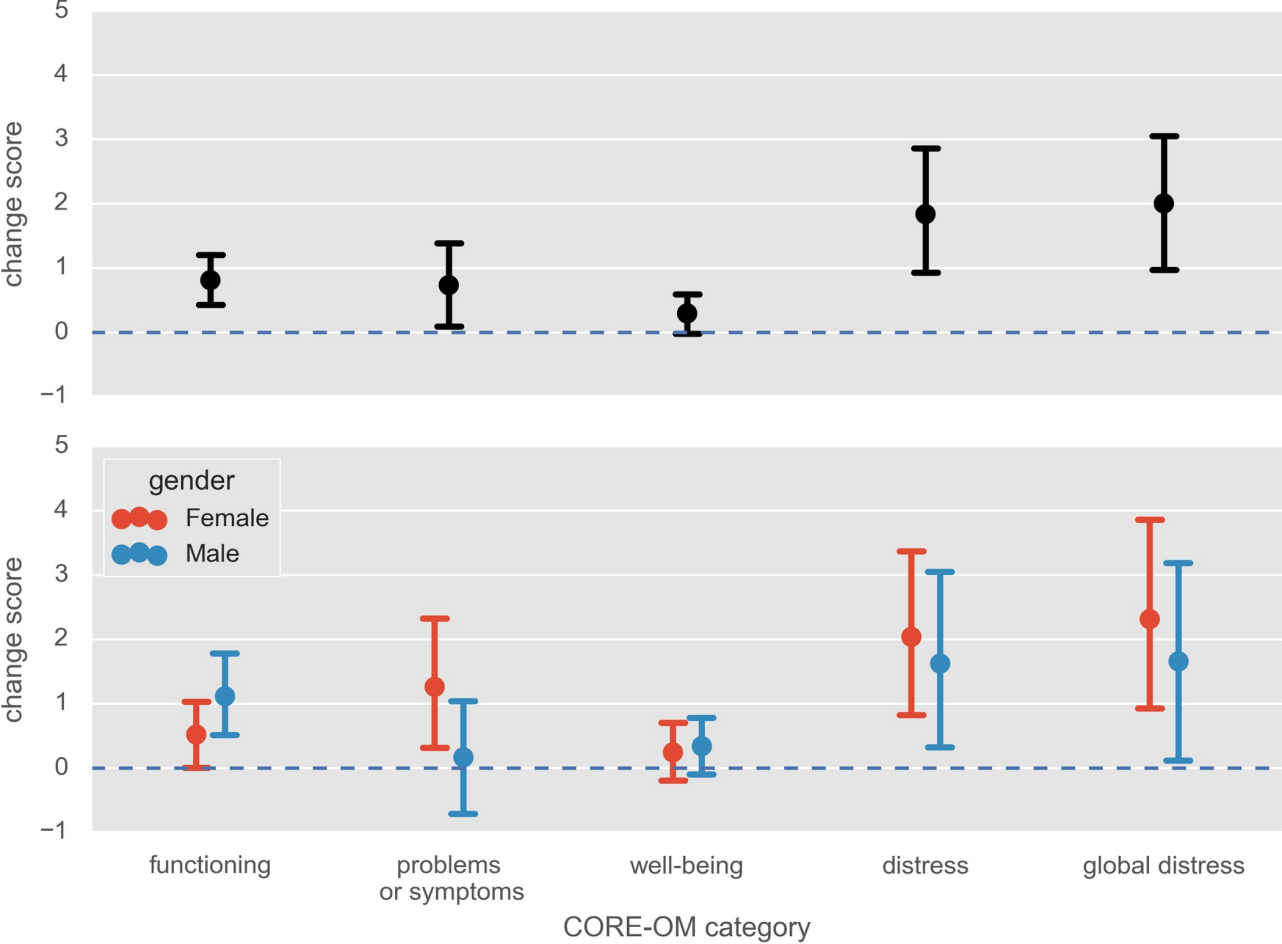
Change scores for dependent variables. Horizontal line at zero indicates no change. Plots represent Mean +/- 2*S.E.

Changes scores were calculated by subtracting post- from pre-recalled-reading state scores of the four dimensions of the CORE-OM (wellbeing, problems or symptoms, functioning, and risk), as well as the compound of these, global distress. Following Connell & Barkham (2007), CORE-OM scores are usually calculated such that increase in distress/negative symptoms are positively coded, so higher scores on the CORE-OM indicate poorer wellbeing. By subtracting post- from pre-recalled-reading scores, we reverse this convention such that in the subsequent analyses, positive change scores indicate increase (and negative change scores indicate decreases) in positive mental states in the post-recalled-reading state compared to the pre-recalled-reading state. To test our hypothesis, we conducted one-way, one-sample *t*-tests for all of these variables, where the null hypothesis is that there is no change (i.e. mean change score of zero). Results are reported in Table 1 and Fig 2, which also breaks results down by gender. In all cases, unadjusted change scores across the whole sample were significantly above zero, indicating that reported positive impact in every factor of the CORE-OM was higher in the recalled post-recalled-reading state than in the pre-recalled-reading state. Applying a Bonferroni correction for multiple tests resulted in significance drop to trend level for problems or symptoms, and to become non-significant for wellbeing—but significance levels remained highly significant for the other variables, including global distress, which is a compound of the other factors, and which was tested first.

**Table 1:** Results of one-sample t-tests on CORE-OM change scores for Study 1.

	*t*	*DF*	*Mean*	*Lower CI*	*p*	*p*_*cor*_ *
*Global distress*	3.679	147	2.003	1.102	< .001	*s*
*Distress*	3.821	147	1.838	1.042	< .001	*s*
*Functioning*	3.766	147	0.811	0.454	< .001	*s*
*Problems or symptoms*	2.2	147	0.733	0.182	.014	*ns*
*Wellbeing*	1.85	147	0.294	0.031	.033	*ns*

In all cases the alternative to the null hypothesis is that the true mean is > 0.

*Adjusted using a Bonferroni correction for multiple hypothesis testing, where *p*_*cor*_ gives whether the result remain significant after being evaluated against *p*_*crit*_, where *p*_*crit*_ = *p/k*, where *k* is the number of hypotheses being tested (*s* = significant; *ns* = not significant).

#### Exploratory analysis

**Fig 3:**
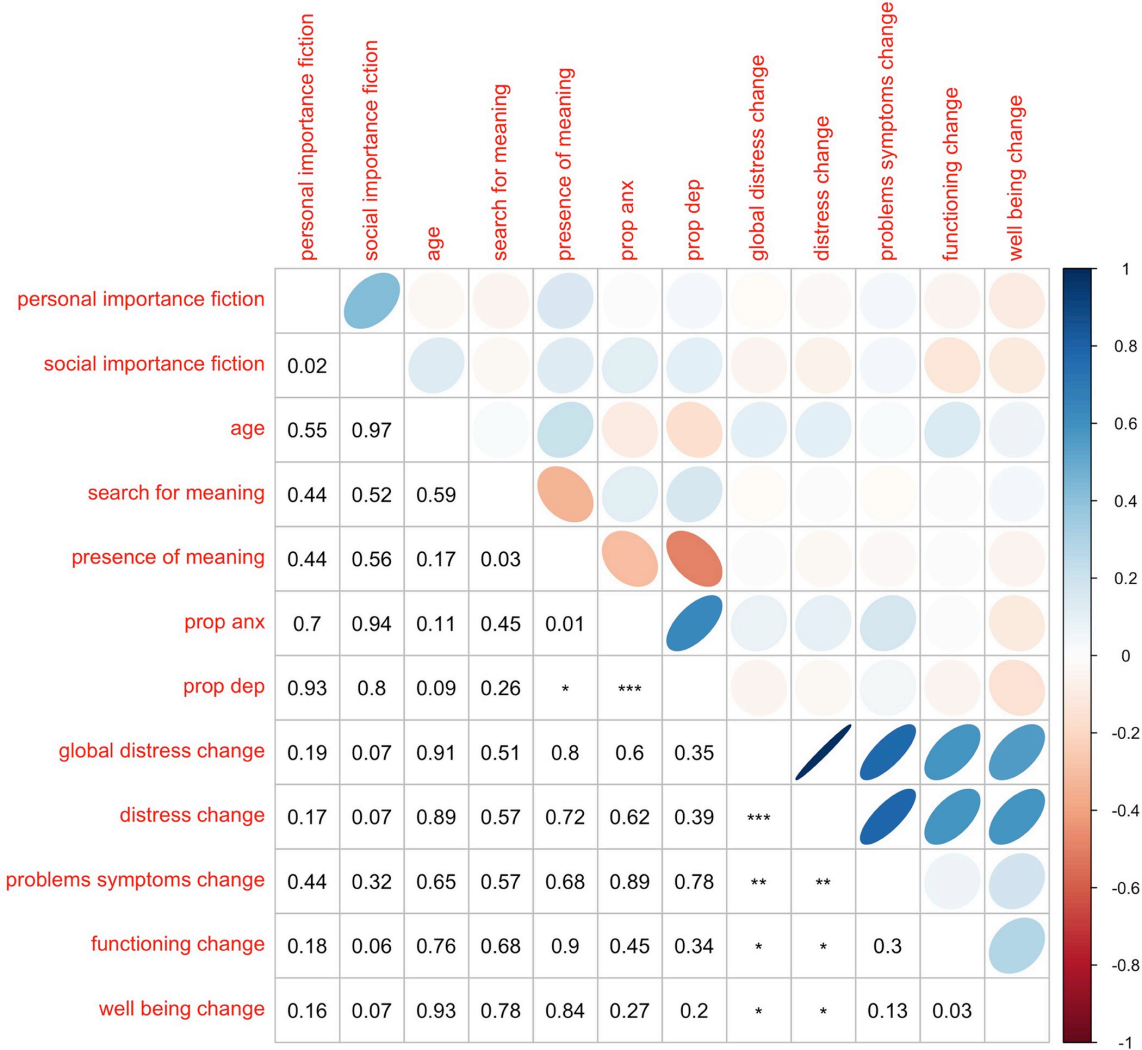
Variable correlation table (*p < .01; ** p < .001; ***p < .0001).

**Fig 4:**
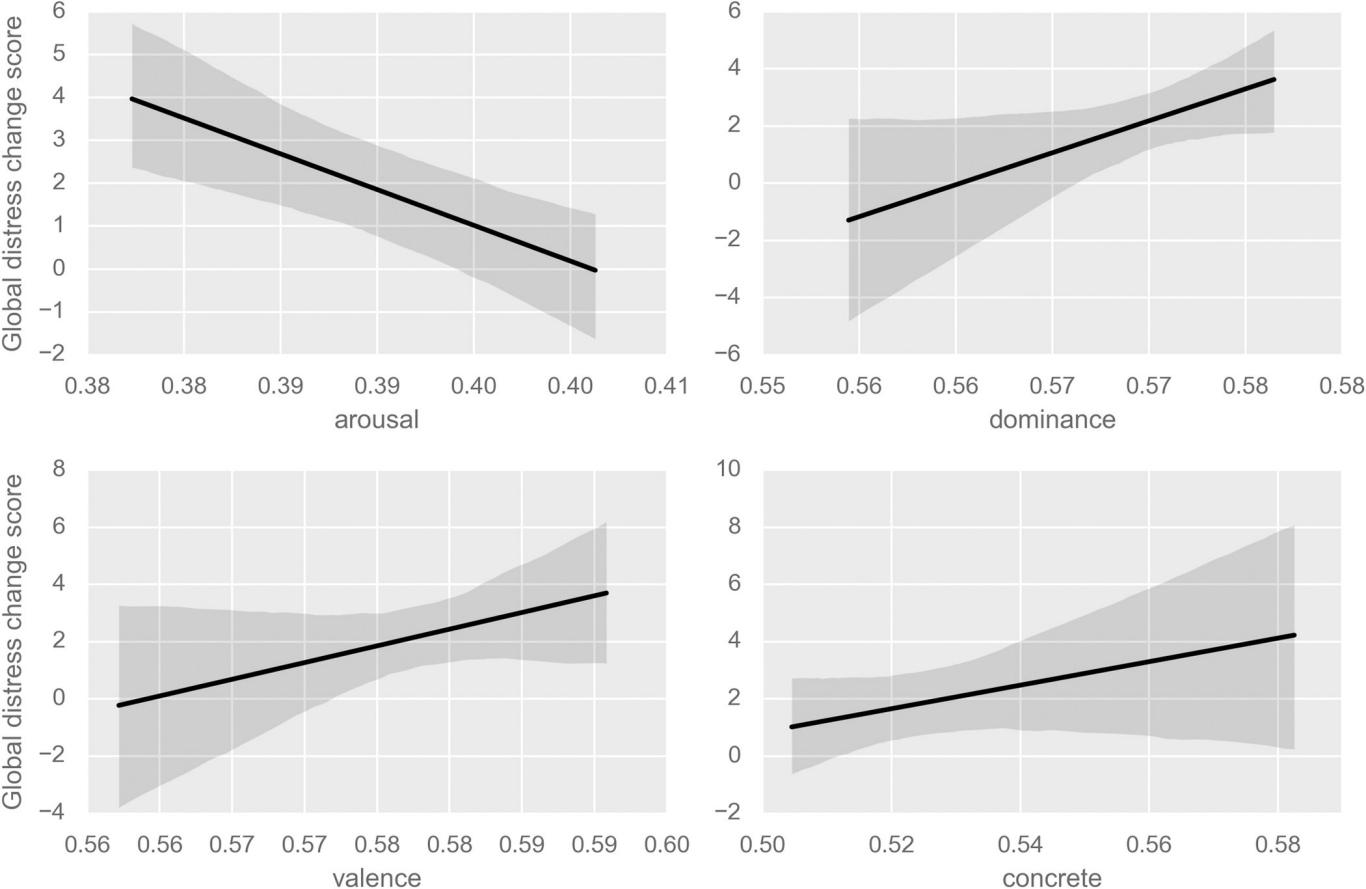
Effects of arousal, dominance, valence, and concreteness on global distress change score in Study 1.

In order to test whether any of the independent or moderating variables related to recalled change in wellbeing, we regressed global distress change on the following variables, as well as all two-way interactions between them: valence, arousal, dominance, concreteness, search for meaning, presence of meaning, propensity to anxiety, propensity to depression, personal importance fiction, social importance fiction, age, and sex. We also allowed intercepts to randomly vary, grouped within participant; this procedure allowed for the effects of grouping variables (like reading the same book) to be accounted for in the analysis. (Participants answered the CORE-OM measure for before and after reading two different books, so change scores on multiple books were grouped within participant.) To reduce the complexity of this model, we then performed a backwards stepwise model selection procedure, where *α*_*crit*_ = .05. As such a procedure increases the likelihood of Type I errors (i.e. false positives), these results should be considered exploratory in nature. The model fitting procedure resulted in the model reported in Table 2 and Fig 3. There are significant effects for valence, arousal, dominance, and concreteness (Fig 4), as well as numerous significant interactions. Specifically, increasing valence, dominance, and concreteness engenders an improvement in the global distress measure, while increasing arousal decreases it. In other words, the more ’sensational’ and negative a text was, the more it was likely to have a negative impact when recalled. As omnibus tests of model fit like *R*^2^ are not possible with multilevel models, we instead report the log likelihood ratio test for our model compared to a model with only the intercept randomly varied by participant. Results are highly significant at *χ*^2^(87.18) = 1629.8, *p* < .001.

**Table 2:** Results of exploratory multilevel model selection procedure in Study 1.

	β	*SE*	DF	*t*	*p*
(Intercept)	-12.002	3.792	218.141	-3.165	0.002
*Valence*	8.744	4.033	220.551	2.168	0.031
*Arousal*	-10.348	3.702	221	-2.795	0.006
*Concreteness*	-13.379	3.436	219.186	-3.894	< 0.001
*Dominance*	-13.5	5.687	220.998	-2.374	0.018
*Search_Mean* *	0.196	0.485	115.799	0.405	0.686
*Prop_Anx* **	0.27	0.655	124.006	0.412	0.681
*Prop_Dep* ***	0.388	0.645	122.109	0.602	0.548
*PI_Fic‡*	-0.086	0.496	129.828	-0.174	0.862
*Age*	0.631	0.522	120.339	1.21	0.229
*gender* ª	0.275	0.505	128.852	0.545	0.587
*Valence x Arousal*	22.993	6.655	220.423	3.455	0.001
*Valence x Concreteness*	15.767	3.642	213.217	4.329	< 0.001
*Valence x Dominance*	20.89	4.656	215.163	4.487	< 0.001
*Arousal x Dominance*	-12.966	5.234	220.755	-2.477	0.014
*Arousal x Prop_Dep*	-3.922	0.793	218.639	-4.943	< 0.001
*Arousal x Prop_Anx*	1.394	0.588	181.786	2.37	0.019
*Concreteness x Prop_Dep*	-2.603	0.646	219.13	-4.033	< 0.001
*Arousal x PI_Fic*	-3.238	1.626	220.255	-1.992	0.048
*Concreteness x PI_Fic*	-3.276	1.302	216.074	-2.515	0.013
*Dominance x PI_Fic*	-3.254	1.242	220.995	-2.619	0.009
*Search_Mean x Age*	1.22	0.557	113.442	2.192	0.03
*Prop_Anx x Age*	1.118	0.507	110.266	2.206	0.029
*Prop_Dep x Sex*	2.751	0.665	127.329	4.14	< 0.001
*Prop_Anx x Sex*	-2.671	0.657	114.961	-4.064	< 0.001

*Search for meaning

** propensity towards anxiety

***propensity towards depression; ‡ personal importance of fiction.

ª*Gender* is sum coded such that the sum of all factor levels = 0, which means significance and estimation of effects should be interpreted as estimates of a given factor level compared to the grand mean between both factor levels. In this case male = -.5 and female = .5, meaning slope estimates should be interpreted as the effect of being female compared to the average of males and females. Multiply the given *b* and *t* values for the effect of *sex* by -1 to arrive at estimates of the effect of maleness compared to the mean of both.

All variables are scaled such that the mean = = 0, so significant "main effects" in the presence of significant interactions should be interpreted as estimating an effect when all other variables are equal to their mean.

### Discussion

Results suggested that being prompted to remember the experience of being immersed in the fictional world can impact positively on psychometrically validated measures of distress, problems or symptoms, and ability to function, with the balance of probability suggesting that this is also the case for well-being. These results provided support for the investigated hypothesis. The exploratory analysis revealed several interactions that may explain this effect, but the model fitting procedure makes inferences unreliable. It did, however, point to VAD values being of interest in subsequent testing. In particular, the analysis indicates that VAD variation should be systematically incorporated into text selection so as to assess any causal role on text impact, particularly with respect to the differential impact of arousal relative to valence, dominance, and concreteness.

### Limitations

The two principal weaknesses of this study were the lack of a control condition and the fact that each answered-on text involved taking the CORE-OM twice in close succession. This means results may not be specific to the recall of fiction and that the pre- and post-intervention measures may not be sufficiently independent of each other.

## Study 2—Impact of prescribed fiction

Our second study evaluated the effect of prescribed fiction. However, it also took cognizance of the limitations of the first study by incorporating a control condition and staggering the first and second administrations of the CORE-OM over several days. In place of the less precise PHQ anxiety and depression battery, we used the Depression Anxiety Stress Scales (DASS) to measure propensity to each condition; we also assessed participants on the ‘Big Five’ personality scale. Another change came in the texts evaluated. Though novels are by far the most common vehicle of narrative fiction, these are too long to be used in an experimental setting. We used short stories instead, which can be read in a single session.

Consistent with the results of the first study, our hypothesis was that reading fiction would lead to improved outcomes on the CORE-OM relative to the control condition However, our expectation was that better diagnostic tools, a larger number of texts, and improved study design would give better insight into the causal mechanisms behind any detected change.

### Materials and methods

#### Participants

Participants were recruited online using TurkPrime with same approval ratings as in Study 1. There were 158 participants in total for the first stage of the experiment; 140 of these completed the second stage. One further participant who entered their sex as ‘other’ was excluded due the difficulty of drawing statistical inferences from a sample of one. This gave a usable *N* of 139 participants (55 females).

#### Procedure

In the first step of the experiment, participants completed the CORE-OM, DASS, ‘Big 5’, and Search for Meaning questionnaires. Six days later, they were contacted and invited to participate in the second stage. On doing so, they were presented with a random story or a control text and asked to read it. Time controls were imposed so that participants could not continue to the next section until enough time had passed for several re-reads of the assigned text. Subsequent to reading, they were asked to complete the CORE-OM again. Participants were paid separately for each arm of the study, with payment for the first arm not requiring completion of the second arm—although participants were strongly exhorted to complete both.

#### Materials

Texts for the test condition were scraped from the American Literature database of 4,000 short stories using a python script written for the purpose. Although described as an American literature website, these stories offered a generic selection from world literature with no obvious bias in favour of American authors. The advantage of this database is that it offered an accessible and independent source of classic literary narratives that were of short duration and out of copyright. One disadvantage is that many of its selections seem dated to the modern reader; another is that the decision to sample the entire space of VAD variation (see below) meant that some children’s stories were included. Though a case could be made for excluding the latter, we felt that methodological rigour was better served by including them.

For the control condition, we used eight Wikipedia articles on neutral topics. There were ‘paint’, ‘dime’, ‘snow’, ‘benzene’, ‘metal’, ‘sand’, ‘ammonia’, and ‘chair’. These were chosen so as to provide an emotionally neutral experience of reading other than fiction. Table 3 summarises the presented texts.

**Table 3:** Texts with VAD + C values and category designation in Study 2.

*title*	*author*	*valence*	*arousal*	*dominance*	*concrete*	*category*
*Passeur*	*Robert W Chambers*	0.56401994	0.36363308	0.55335282	0.67776	V↓A↓D↓
*A Reminiscence of Dr*. *Samuel Johnson*	*H P Lovecraft*	0.57243821	0.38158032	0.55923954	0.502666	V↓A↓D↓
*A Deal in Wheat*	*Frank Norris*	0.5652027	0.37610304	0.55919088	0.570482	V↓A↓D↓
*The Servant*	*St Semyonov*	0.57411894	0.37531755	0.56756608	0.495811	V↓A↓D↓
*Beyond the Door*	*Philip K Dick*	0.58479412	0.37076618	0.57466029	0.56258	V↓A↓D↑
*Her Lover*	*Maxim Gorky*	0.58770519	0.37531826	0.57856156	0.533824	V↓A↓D↑
*Conversation On Conversation*	*Harriet Beecher Stowe*	0.58424047	0.38201261	0.58708026	0.448569	V↓A↓D↑
*About Barbers*	*Mark Twain*	0.55923026	0.3664693	0.57220833	0.601898	V↓A↓D↑
*Accessory Before the Fact*	*Algernon Blackwood*	0.54915084	0.38405063	0.55443565	0.527732	V↓A↑D↓
*A Ghost*	*Lafcadio Hearn*	0.57931498	0.3883423	0.55880381	0.44847	V↓A↑D↓
*The Sniper*	*Liam O’Flaherty*	0.49607143	0.41358135	0.52722817	0.647572	V↓A↑D↓
*A Child of the Rain*	*Elia W Peattie*	0.56482143	0.38697719	0.55323795	0.599676	V↓A↑D↓
*How It Happened*	*Sir Arthur Conan Doyle*	0.58701014	0.38886824	0.58472973	0.568585	V↓A↑D↑
*A Case of Desertion*	*WW Jacobs*	0.57133259	0.39745982	0.57548884	0.581466	V↓A↑D↑
*On Nikolai Vasilievich Gogol*	*Prosper Merimee*	0.58309634	0.39452447	0.57794919	0.46809	V↓A↑D↑
*When Papa Swore in Hindustani*	*P G Wodehouse*	0.58761646	0.38736607	0.58250194	0.510109	V↓A↑D↑
*Hickory*, *Dickory*, *Dock*	*L Frank Baum*	0.59289934	0.37115312	0.56478261	0.622011	V↑A↓D↓
*Jimmy Scarecrow’s Christmas*	*Mary E Wilkins Freeman*	0.58961145	0.37479367	0.56200904	0.614698	V↑A↓D↓
*The Tale of Benjamin Bunny*	*Beatrix Potter*	0.6006191	0.35762677	0.5694487	0.673203	V↑A↓D↓
*Mother Bear’s Call*	*Harriet Prescott Spofford*	0.59986111	0.37614953	0.56063534	0.603165	V↑A↓D↓
*The Fable of the Two Mandolin Players*	*George Ade*	0.62161045	0.36325728	0.59596561	0.529351	V↑A↓D↑
*An Ideal Family*	*Katherine Mansfield*	0.60513201	0.37541529	0.5722896	0.567283	V↑A↓D↑
*A Bread and Butter Miss*	*HH Munro Saki*	0.61133266	0.37768548	0.5849254	0.532385	V↑A↓D↑
*A Piece of Red Calico*	*Frank Stockton*	0.60997496	0.36763867	0.58786017	0.582481	V↑A↓D↑
*A Story Which Will Never Be Finished*	*Leonid Andreyev*	0.59818631	0.39427017	0.56404866	0.568907	V↑A↑D↓
*A Dark Brown Dog*	*Stephen Crane*	0.59536987	0.40768359	0.56069924	0.580252	V↑A↑D↓
*A May Evening*	*Nikolai Vasilievich Gogol*	0.60881981	0.38584608	0.57000332	0.588806	V↑A↑D↓
*A Foolish Man*, *Philosopher*, *and Fanatic*	*William Dean Howells*	0.59467491	0.39385501	0.56754315	0.542944	V↑A↑D↓
*One Christmas at Shiloh*	*Paul Laurence Dunbar*	0.60152548	0.3889876	0.57955062	0.53143	V↑A↑D↑
*Christmas*	*Washington Irving*	0.64221262	0.39518159	0.59554478	0.497714	V↑A↑D↑
*After the Race*	*James Joyce*	0.62142327	0.39459004	0.59329517	0.564705	V↑A↑D↑
*Blessing of a Good Deed*	*Mary Roberts Rinehart*	0.60862879	0.39835795	0.58189583	0.50162	V↑A↑D↑
*paint*	*Wikipedia*	0.567664	0.349257	0.576624	0.614587	*control*
*dime*	*Wikipedia*	0.593733	0.366514	0.580905	0.609678	*control*
*snow*	*Wikipedia*	0.592497	0.361729	0.553352	0.615662	*control*
*benzene*	*Wikipedia*	0.553296	0.368692	0.550811	0.55659	*control*
*metal*	*Wikipedia*	0.563479	0.369914	0.574286	0.579968	*control*
*sand*	*Wikipedia*	0.584417	0.349259	0.557503	0.635833	*control*
*ammonia*	*Wikipedia*	0.537697	0.366696	0.551779	0.564316	*control*
*chair*	*Wikipedia*	0.574889	0.329085	0.573896	0.652029	*control*

Arrows designate whether a story is higher or lower than the corpus mean for that category.

#### Independent variables

All texts were processed and assigned a VAD+C score using the spaCy function created in Study 1. With a view to providing a balanced sample, each story was also assigned a category designation that showed where it sat in the three-dimensional space of VAD variation. The eight categories used corresponded to whether the V, A, and D measures associated with each story were higher or lower than the zero-centred mean of each dimension for the whole corpus. With a view to maximising variance, the Euclidean distance associated with each story was taken and used to rank it relative to other stories in the same category, with the story furthest from the origin being given a rank of 1. A second constraint was imposed by word count. Allowing that each story would need to be read several times, this meant an estimated reading time of 1 minute for 238 words gave an exposure time of 18 minutes for three readings of a 1,500 word story [49]. This giving an upper limit, we placed the lower limit at 600 words so as to avoid acutely dissimilar experiences of reading time across participants. Within the word count constraint, we selected the four stories in each of the eight categories that had the greatest Euclidean distance, giving 32 stories in total. Combined with the eight control condition articles, this gave 40 test items. (The entire dataset of 4k processed stories can be downloaded from X.)

#### Dependent and moderating variables

*CORE-OM and SM*. Following the procedures established in Study 1, the CORE-OM and Search for Meaning scales were used to measure therapeutic impact and propensity to search for meaning respectively. See previous study above for details of these questionnaires.

*Depression and anxiety*. Instead of the PHQ4 measure for anxiety and depression that we used in Study 1, we chose here to use the Depression Anxiety Stress Scales (DASS) questionnaire [50]. Though the DASS and the PHQ correlate strongly with each other, the DASS offers a more probing inventory of 21 questions that subdivide into three factors of anxiety, depression, and stress. As such, our view was that the DASS would perform greater explanatory work were propensity towards depression or anxiety discovered to have an effect.

*Big 5 personality scale*. The Big 5 model of interpersonal variation suggests that human beings differ from each other with respect to the traits of openness to new experience, conscientiousness, extraversion, agreeableness, and neuroticism [51, 52]. There is a mixed literature on the relationship between the Big 5 and cultural preferences. Djikic, Oatley, & Carland (2012) [53], for instance, suggest that exposure to fiction can cause changes in self-assessment with respect to the Big 5; other studies argue that personality, far from being shaped by cultural objects, determines the cultural objects that are found to be engaging and how they are engaged with [54–56]. Give the plausibility of claims that personality variation may moderate cultural taste (and vice versa), we included the 44-item inventory for measuring the five personality factors from John & Srivastrava [57].

### Results

As with Study 1, change scores were calculated on the CORE-OM by subtracting scores at the different time points. Change scores on the test condition were then compared with scores in the control condition using an independent samples t-test on *global distress*, *problems or symptoms*, *distress*, *well-being*, and *functioning*. No statistically significant differences were detected on any dependent variable (Table 4). Subsequent tests that compared responses in each text category with the control condition also yielded no significant results.

**Table 4:** Results of independent samples t-tests on CORE-OM change scores for test and control conditions in Study 2.

	*t*	*DF*	*Mean*	*Lower CI*	*p*
*Global distress*	0.100	60.58	2.003	-0.15	.92
*Distress*	0.137	81.06	1.838	-0.11	.89
*Functioning*	0.298	62.66	0.811	-0.18	.76
*Problems or symptoms*	-0.195	68.28	0.733	-0.24	.84
*Wellbeing*	-0.083	62.99	0.294	-0.15	.93

### Discussion

The lack of significant results here challenges the outcome of Study 1. However, the grounds of the challenge are not clear. One possibility is that the design of Study 1 created an artefact and the detected effects were not real. A second is that the constraints imposed by VAD sampling and word limits created an ecologically unrealistic sample of texts (i.e. the texts, taken collectively, are not a good representation of fiction). Thirdly, it could be reasonably argued that the CORE-OM outcome measure—designed for measuring face-to-face therapeutic interventions for distressed individuals—is inappropriate to the measurement of subtle changes in affective and cognitive state brought about by reading fiction. Finally, it may be that prescribing fiction independently of individual preferences nullifies any therapeutic impact it may have. Testing the impact of chosen fiction using more sensitive measures than the CORE-OM allows us to adjudicate between these outcomes by giving a second methodological contrast to Study 1 that avoids prescribing fiction to participants and is not subject to the same exacting sampling constraints.

## Study 3—Impact of choosing fiction

If fiction has a therapeutic impact, it may be that this impact is partly delivered by the act of choosing the fictional text. That is, independently of the character of the text itself, individuals may respond to it more positively when the experience of reading is felt to be congruent with an autonomous choice. Support for this idea comes from the large literature documenting how individuals denied a choice in an activity experience less hedonic return relative to individuals allowed to exercise a choice [58–60]. In the specific area of cultural choices, for instance, Lewis [61] shows that the same TV programmes are enjoyed more by viewers who choose them over viewers who are involuntarily exposed to them, an effect that is (with some qualifications) also visible in advertising [62, 63].

Our third study evaluated whether these ideas can be extended to fiction. It did this by reproducing the two-arm design of Study 2, but instead of assigning fiction, it allowed participants to choose the texts they wished to read. However, though we followed the overall design of Study 2, we changed several of the dependent variables. Most significantly, we used the Profile of Mood States (POMS) measure to capture affective change between the first and the second timepoints of the study [64]. We also swapped the CORE-OM for the GP-CORE (a subset of the CORE-OM questions that is intended for use with a general population instead of a clinical one) and used the GP-CORE score as a trait rather than a state measure. These changes are explained in more detail below.

### Materials and methods

#### Participants

Participants were recruited online using TurkPrime, using the same approval ratings as the previous two studies. There were 219 participants in total for the first stage of the experiment; 154 of these completed the second stage. One participant who entered their sex as ‘other’ was excluded due the difficulty of drawing statistical inferences from a sample of one. Two further participants were excluded on account of being mistakenly served the blurb text for a Gabriel García Márquez story instead of the story itself. This gave a usable *N* of 151 participants (69 females). As the duration of the task assigned to each participant varied widely, a three-tier payment structure was adopted, such that participants who chose longer tasks were paid more.

#### Procedure

In the first step of the experiment, participants completed the GP-CORE, Search for Meaning and Profile of Mood States (POMS) questionnaires. Six days later, they were contacted and invited to participate in the second stage. In the test condition, they were invited to select a short story from a dropdown list and asked to read it, where the list text gave the name of the story and the name of the author. Time controls were imposed so that participants could not continue to the next section until enough time had passed for several re-reads of the selected text. In the control condition, they were randomly assigned one of the eight Wikipedia articles that made up the control texts in Study 2. Subsequent to reading, they were asked to complete the POMS questionnaire again. Participants were paid separately for each arm of the study, with payment for the first arm not requiring completion of the second arm.

#### Materials

Texts were taken from the American Literature database of 4,000 short stories used in Study 2. Unlike Study 2, however, there was no requirement to balance the selection across VAD+C. This meant that the primary considerations were word count and the challenge of loading the story text into the survey software, which required manual text formatting using html tags. In the interests of realistic reading times, word count was capped at 20k words and the 32 stories from Study 2 were increased to 67 so as to provide greater story choice. The control texts were once more the Wikipedia articles for ‘paint’, ‘dime’, ‘snow’, ‘benzene’, ‘metal’, ‘sand’, ‘ammonia’, and ‘chair’. Participants from Study 2 were excluded from Study 3.

#### Independent variables

The hypothesis informing this study was that choosing a fictional text would have a stronger impact on well-being than reading a randomly assigned alternative text. For this reason, the independent variable was categorical, and consisted of whether the participant chose (or did not choose) the text they were exposed to. In the test condition, this involved being exposed to a voluntarily chosen piece of fiction; in the control condition, they were randomly exposed to a Wikipedia article. As Study 2 already indicates that prescribing fiction does not seem to have a therapeutic effect, any positive result in the test condition in the present study favours the hypothesis that choosing a text has an impact on well-being. We acknowledge that this leaves undecided the question of whether a similar effect would also be achieved by choosing a non-fiction text. However, as our aim was to establish the result of choosing fiction relative to prescribing fiction compared to the same control, the best procedure was to retain the control texts from Study 2.

#### Dependent and moderating variables

In contrast to Study 2, we used the POMS inventory rather than the CORE-OM as the dependant variable. On consideration, we felt that the CORE-OM, as an inventory designed to test structured therapeutic interventions, may not have the sensitivity to capture improvements to well-being that did not reach the threshold of clinical significance. By contrast, the POMS inventory is explicitly designed to capture transient changes in mood states that load onto the dimensions of tension, depression, anger, vigour, fatigue, and confusion [64]. In the interests of brevity, we used the reduced 39-item scale validated in Grove & Prapavessis [65] rather than the original 64-item questionnaire. Questions ask participants to quantify how they are feeling at the time of testing by presenting a mood word—‘Tense’, ‘Sad’, ‘Energetic’, etc.—and requiring them to circle an option on a scale that runs from ‘Not at all’ to ‘Extremely’. Several precedents exist for using POMS to capture affective and cognitive change following interventions designed to improve well-being [66–68].

Paralleling this movement away from measures designed to capture clinical variation, we took the GP-CORE as a moderating variable instead of the DASS. This is a subset of 14 questions from the CORE-OM that are selected for use in mental health assessment outside of clinical settings [69]. The chief difference is that extreme questions like ‘I have been physically violent to others’ or ‘I have felt panic or terror’ are omitted in favour of questions that pick out non-clinically relevant emotional variation.

The other included moderating variable, the Search for Meaning measure, was included due to it predicting receptivity to literary effects in Carney & Robertson [47]. The Big 5 personality scale was not used as analysis of Study 2 results showed no significant variation in responses across the five personality factors.

### Results

Change scores were calculated for POMS on two dimensions: total mood disturbance (TMD) and esteem-related affect (ERA). The first is the sum of the scores on the negatively valent dimensions minus the sum of the positively valent ones; the second is the specific subset of questions that pertain to positive mood states. An independent samples t-test was used to compare change scores between the test and control conditions. No statistically significant results were recorded on either dimension (TMD: *t* = -0.382, *DF* = 48.01, *lower CI* = -9.32, *p* = .703; ERA: *t* = -1.04, *DF* = 56.53, *lower CI* = -2.09, *p* = .301).

### Discussion

The lack of statistically significant differences between groups in this study challenges the hypothesis that choosing fiction impacts on wellbeing. Given that this matches the results achieved for prescribing fiction, the bigger question it raises concerns whether any positive results can be expected from direct exposure to fiction. It may be, for instance, that fiction needs to be cognitively or emotionally processed before positive effects can be accessed. This means that the testing programme needs to incorporate these dimensions if fiction is to be properly assessed for its impact on well-being.

## Study 4—Impact of discussing fiction

One of the most common ways in which fiction is processed is through group discussion. In the first instance, most comprehensive education programmes will include a component that involves reading and discussing fiction, meaning that even individuals who do not enjoy fiction will have experience discussing it. Beyond educationally mandated discussions of fiction, books clubs provide popular forums in which like-minded individuals can discuss named titles. Typically, such groups are independent of each other, but they are occasionally coordinated in a top-down way by public libraries or TV shows. Finally, evaluations of fiction by academic researchers and professional journalists constitute a type of collective cultural discussion that individuals participate in to greater and lesser degrees, just as groups of friends who associate for social reasons may discuss fiction as part of general conversation.

Our fourth study evaluated the impact on mood and emotion of discussing book-related topics (mostly fiction) relative to the discussion of non-book-related topics. We chose to use the VAD word norms explored in the previous studies to measure mood and emotion. However, we supplemented this with an ‘absolute word’ measure, which has previously been used to assess linguistically mediated mental distress. In practice, this involved testing four claims.

1. *Valence*: Discourse on books should be more positively valent, on average, than discourse on other topics. This follows directly from bibliotherapeutic claims that reading has positive impacts on well-being. Though this does not imply that discourse on reading will be the most positively valent of all surveyed discourse, it should score significantly higher than a random selection of other topics.
2. *Arousal*: Discourse on books should be lower in arousal, on average, than discourse on other topics. While it is certainly true that high arousal is consistent with both positive and negative experiences, states of high physiological excitement are particularly associated with stress [70, 71]. Thus, if discourse about books has a therapeutic effect, it is to be expected that it would exhibit lower arousal than other topics of discussion.
3. *Dominance*: The conviction that one is the author of one’s own actions is often cited as a feature of psychological well-being [72–74]. Any positive impact of discourse on books on well-being should therefore be characterised by higher mean levels of dominance, which measures how in-control a stimulus makes its experiencer feel. Thus, we predict that dominance levels should be higher in book-related topics than in other topics.
4. *Absolute words*: Al-Mosaiwi and Johnstone [35] argue that the use of what they call ‘absolute words’ is a linguistic covariate of anxiety, depression, and suicidal ideation. These are words that express magnitudes or probabilities without nuance or qualification, such as ‘always’, ‘totally,’ or ‘entire’. The supposition is that mental distress results in over- or underestimation of threats and rewards and the use of absolute words characterises this. We therefore predict that that the probability of encountering absolute words should be higher in non-book-related subreddits.

Given that one of the present authors was already involved in a long-term, in-person study that evaluated the impact of reading and discussing fiction [37], we chose here to focus on online discussions of fiction from the Reddit social media platform. While it is only fair to acknowledge from the outset that how people talk about a topic online may differ from offline interactions, it is unlikely to be wholly dissimilar. Moreover, given the role of social media in present-day social interactions, any results will be of equivalent importance to those from in-person studies.

### Materials and methods

#### Participants

Participants were not directly recruited. As per the procedure outlined below, they were selected when Reddit was accessed using the Reddit API in accordance with specific search criteria. This produced 286 participants. All scraped data were already in the public domain, but Reddit usernames were nevertheless hashed so as to protect the identity of users who may have been using their actual name.

#### Procedure

Reddit is an open platform that allows users to create and participate in themed forums; its functionality allows for users (‘redditors’) to post original content, links, or other media and comment on it. Specific comments and posts can be voted up or down by individual users, with upvoted content remaining visible for longer. At the time of writing, Reddit has over 330 million users and approximately 180 thousand active subreddits, with the latter covering every major articulation of human activity. What makes Reddit useful in the present connection is that it can be accessed via an API that makes it possible to quickly and efficiently aggregate large bodies of text data. This makes it ideal for comparing the different ways in which people talk about different activities.

Using the Python Reddit API Wrapper (PRAW), the authors wrote scripts for extracting content from Reddit. For any specific subreddit, the scripts extracted the most upvoted submissions. With respect to an individual submission, the relevant script scraped all the comments associated with that submission. Two scripts dealt with individual redditors: one of these extracted the comments of a particular redditor on other redditors’ submissions in order of most upvoted comments; the other pulled their own submissions in order of upvotes. The scrape was initiated by scraping the top-rated 1,000 submissions in the books subreddit, with the ratings attaching to the number of upvotes received. The authors of these posts were identified, and all of their other submissions and comments were extracted up to a maximum value of 1,000 items. Each script generated a dataframe with the same columns—namely, ‘text’, ‘datetime’, ‘score’, ‘title’, ‘subreddit’, ‘type’, and ‘redditor’. Where a given script did not return a value for column field, a ‘NaN’ (‘not a number’) value was recorded. Using the same columns allowed for the output of one script to be easily concatenated with the results of every other. In all cases, content extraction was capped by PRAW rate limits, which return a maximum of 1,000 items for any Reddit listing and only allow a finite number of API calls in a given session.

Data were extracted on the 20^th^ of May 2019. This generated a dataset of 251,403 observations. Of these, 22,803 were generated by the AutoModerator Reddit bot and were removed, as were occasional duplicate entries. The result was a dataset of 228,600 items, spread across 5,942 subreddits.

#### Experimental variables

Clean text and VAD values were extracted using a spaCy process function built in Study 1; an absolute words metric was also calculated for each text item. As processed texts were on average relatively short (*M* = 40.14 words), this meant that a taking a mean across words for each of valence, arousal, and dominance gave an accurate measure of the emotional components of each item. (This is because longer linguistic samples tend to regress towards the mean VAD of English as a whole.). However, it should be noted that there was high variability in item length (*SD* = 94.45), so internal VAD variation in longer texts may not have been captured. The absolute word metric was calculated by determining the probability of a randomly selected word being an absolute word for every text item.

With a view to distinguishing between books-themed subreddits and non-book-themed subreddits, the ‘subreddit’ column was analysed. Using string matching methods, any subreddit that contained ‘book’ or ‘fiction’ in its name was identified. Manual examination of these identified 30 subreddits that were directly concerned with the discussion of fiction or with reading generally. These were distinguished from other subreddits by adding a further ‘genre’ column to the dataframe that grouped books-related subreddits and non-book-related subreddits (here the word ‘genre’ is used in the loosest possible sense as a grouping variable). This gave 198,464 non-reading related items relative to 30,136 reading-related items.

### Results

**Fig 5:**
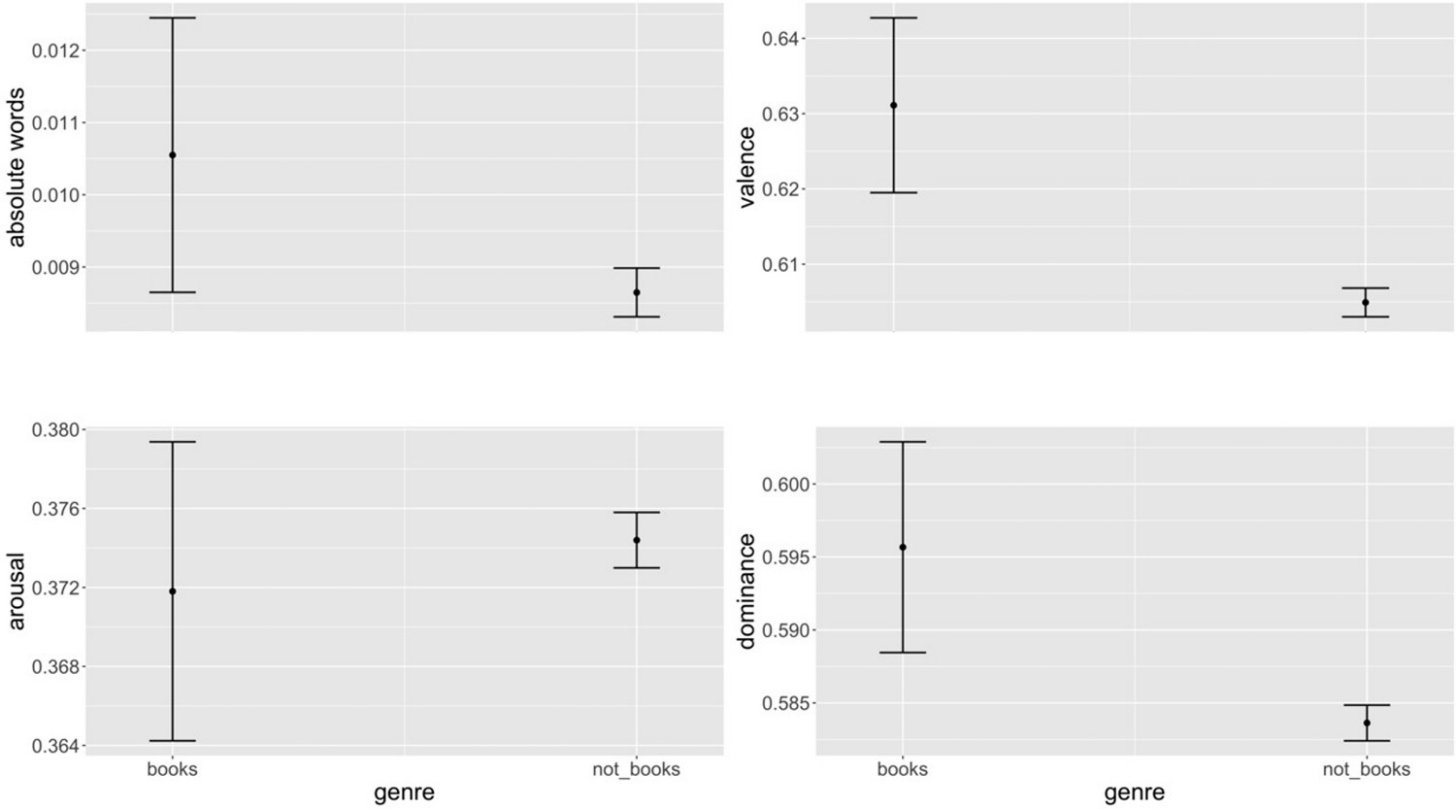
Predicted marginal means for the effect of book-themed and non-book-themed discussion on valence, arousal, dominance, and absolute words.

Valence, arousal, dominance, and absolute words were regressed on multilevel mixed models with random intercepts and random slopes for redditor and subreddit (this allowed for the effects of autocorrelation within multiple posts by the same user and within multiple posts in the same subreddit). Fixed effects were included for whether or not the genre was related to books. This initial model failed to converge so the random slopes stipulation was removed in favour of a model with random intercepts only, which reached convergence. Results are summarised in Table 5, but genre had a statistically significant impact on all variables with the exception of arousal. Effects were in the predicted directions for valence, arousal, and dominance, but in the opposite direction for absolute words (Fig 5). As Study 5 also used the same variables as this study, a Bonferroni correction was made to allow for multiple comparisons on the same dependent variable; this had the effect of making absolute words statistically non-significant.

**Table 5:** Results of mixed linear models for absolute words, valence, arousal, and dominance regressed on genre (book related subreddit vs non-book-related subreddit) for Study 4.

*DV*	*IV*	β	*SE*	*df*	*t*	*p*	*p*_*corr*_
arousal	(Intercept)	0.375	0.003	1650	96.276	< .001	*s*
	books	-0.0025	0.003	1577	-0.677	0.49	*ns*
valence	(Intercept)	0.6311	0.005	1808	106.552	< .001	*s*
	books	0.0219	0.005	1746	4.448	< .001	*s*
dominance	(Intercept)	0.597	0.003	1584	161.732	< .001	*s*
	books	0.012	0.003	1525	3.289	< .01	*s*
absolute words	(intercept)	0.01	0.0009	669.9	10.887	< .001	*s*
	books	0.001	0.0009	649.5	1.971	0.049	*ns*

Slopes estimated with respect to book-related subreddit classification.

### Discussion

Of the three VAD variables, dominance is probably the one most explicitly connected with well-being. Valence and arousal, though obviously central to affective experiences, are nevertheless more directly associated with changes in physiological states. Dominance, by contrast, attaches to the sense of agency: it relates to how autonomous a stimulus makes a person feel. As attested in the literature, autonomy is a signal of both positive mental health and, where mental illness has previously supervened, recovery from such illness [75, 76]. In one formulation, ‘recovery is a journey, characterized by a growing sense of agency and autonomy’ [77]. Thus, the significant result for dominance counts as a useful item of evidence in favour of the view that discussing books can have a positive effect on mental health. What is less clear is why discussing books should foster dominance to a greater degree than other topics. Though we did not disambiguate between fiction and books more generally in this study, the predominance of fiction in the data means that the answer may lay in the effects of narrative on identity construction. Several theorists argue that individuals frame their personal and collective identities using narrative modes of thinking [78–81]; if so, discussing recently consumed fictional narratives may activate a high dominance stance.

The significant result for valence also points to the value for mental health in discussing books. That a positive frame of mind should be associated with mental health is unsurprising; where the puzzle arises is why books should systematically impact on valence. Many books, whether factual or fictional, deal with unpleasant subject matter and challenging experiences, which would lead one to expect wide variation in valence in discussions of these books. Instead, the discussions have a positive valence that is significantly higher than the mean for other topics. It would seem, therefore, that reading has the effect of allowing readers to access and talk about low valence content in a way that is not itself negatively valent.

It is worth noting that this effect of valence replicates results for in-person, group discussions of fiction in Troscianko, Carney, and Holman [82], which show that low valence in a text does not mandate that the discussions of text are recalled as being unpleasant. It also aligns with a long-standing view that assigns a cathartic or functional value to being exposed to third-party experiences, for example via fiction. Aristotle first mooted the idea that drama has the effect of purging powerful emotions in the audience [83]. Twenty-first century scholarship has taken up this idea, with Glavin & Montgomery (2017) hypothesising that fictional worlds make anxiety inducing experiences salient while removing their threatening immediacy, thereby allowing them to be processed in a safe way. Similar ideas are also advanced for fiction by Troscianko [17] and Koopman [84]; Khoo & Oliver [85] make equivalent claims for cinema. Moving on to the functional advantages of representations of others’ experiences, Pinker [86] argues for the view that fiction may have evolved to allow for the offline processing of challenging scenarios. Clasen [87, 88], takes up this idea with respect to negatively valent content in genres like horror; his claim is that the enjoyment of this disturbing content can be explained as an incentive for processing evolutionarily relevant threats like predation. Thus, there are several mechanisms that can explain why discussing books, in particular, should be associated with positive valence when their content may often have a negative valence.

**Fig 6:**
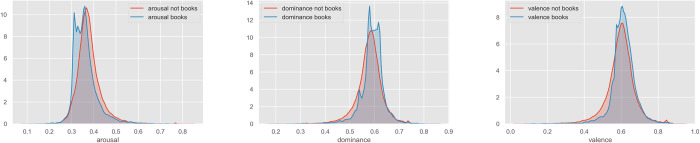
Two-sample Kolmogorov-Smirnov tests for differences in distribution shape significant at the *p* < .001 level (test statistics: 0.2, 0.17, 0.14).

The result on arousal *prima facie* challenges the hypothesis that discussing books improves mental well-being. It is notable, however, that the distribution of values found here for arousal is strongly bimodal—an effect that is also visible for dominance and (to a much smaller extent) for valence (Fig 6). This suggests that books-related discussion does not exhibit uniform variance, but that people tend to use language that peaks either higher or lower than the mean for arousal and dominance. For dominance, the lower peak is still greater than the mean dominance for non-books discussion; for arousal, the higher peak is close to the non-books mean, while the lower peak gives a smaller value than the non-books mean. It would seem, therefore, that, in books-themed discussion there is a latent variable systematically pulling discourse into high-dominance, low-arousal and into low-dominance, high-arousal states. As much of books-related discussion is evaluative, we conjecture that this hidden variable is the quality of a book, with low quality books driving the low-dominance, high-arousal responses and high-quality books informing the high-dominance, low-arousal responses. We recognise, however, that these kernel-density estimate plots do not account for the autocorrelation of errors in the data, and thus make Type I errors of interpretation more likely.

Our hypothesis is challenged by the result on absolute words, even if this effect becomes non-significant after correction for multiple comparisons. We note, however, that evaluative language uses many of the words in the absolute word lexicon, so it may be that absolute words and evaluative language are both forms of assessment applied to different things (i.e. cultural objects and one’s own mental state). Regardless, it does suggest caution in interpreting the overall set of results as being supportive of the idea that discussing books improves wellbeing.

### Limitations

One limitation of this study is that it did not systematically account for variation within books discussed. Though the majority of book-themed discussion concerns fiction, we did not control for how much. As our overall hypothesis concerns how fiction impacts on wellbeing, a more precise focus on fiction is needed. A second limitation is that the high variability in the length of reddit posts means that taking a per-item VAD average may conceal VAD variation within items. Limiting the length of items is needed to prevent this.

## Study 5—Impact of discussing literary and non-literary fiction

One assumption of creative bibliotherapy is that any therapeutic efficacy fiction may have will scale with the quality of that fiction. That is, literary fiction will have a more salutary effect than non-literary fiction. This assumption is challenging to test because ‘literary’ is not an objective designation: it usually functions to identify what a historically shaped interpretive community nominates as literature rather than any feature of the text itself [89]. In Studies 1–3, this was side-stepped by using classic texts that have already been selected by critical opinion as exemplars of literature. The fact that two of these three studies showed no change means we must question the claim that the experience of reading literature has therapeutic effects. However, Study 4 did find effects, and gave evidence to think that there may be a latent variable to do with quality affecting discussions of books. This possibility justifies a study that evaluates the differential impact on wellbeing of literary fiction relative to non-literary fiction in a context that relates to community discussion.

Our fifth study did this by evaluating fiction-themed discourse on Twitter. Specifically, we compared the VAD and absolute words profile of the language surrounding best-seller fiction relative to that surrounding fiction that has won or been shortlisted for a literary award. There were three reasons for choosing to use Twitter as the source of data for this study. The first is that it gives access to the judgments of an interpretive community in an accessible way. Literary awards are explicitly offered as the outcome of informed critical opinion, so winners of these awards can with reasonable confidence be identified as ‘literary’ by the standards of present-day judgment. This contrasts with best-seller lists, which capture titles that are popular without necessarily being literary (allowing that the two can sometimes coincide). The second reason for using Twitter responses to literary awards is that it gives access to opinions on long-form contemporary fiction. Two limitations of earlier studies were that texts were required to be older than 70 years (due to copyright law) and, for Study 2 and Study 3, needed to be short enough to be presented in an experimental setting. As the effect of both limitations was to exclude much of the fiction read by present-day audiences, it challenges the relevance of these studies for contemporary readers. By contrast, Twitter responses to literary rewards and best-sellers directly connect with longer instances of contemporary fiction. Our third reason for using Twitter is that Twitter responses are limited to 280 characters. Tweets do not allow much scope for emotional equivocation, so they resolve the problem of VAD variability presented by long Reddit posts in Study 4.

With respect to hypotheses, we make the same predictions as made in Study 4, but with ‘book-related’ and ‘non-book-related’ replaced by ‘best-seller’ and ‘literary award’. That is, we predict that posts about books on the literary award category will (1) be higher in valence, (2) be lower in arousal, (3) be higher in dominance, and (4) have a lower probability of featuring absolute words.

### Materials and methods

#### Participants

Participants were not directly recruited. As per the procedure outlined below, they were selected when Twitter was scraped in accordance with specific search criteria. This produced 129,134 participants. All scraped data were already in the public domain, but Twitter usernames were nevertheless hashed so as to protect the identity of users who may have been using their actual name.

#### Procedure

We identified six well-known literary awards for contemporary fiction: the Booker International, the Walter Scott Prize, the National Book Critics’ Award, the Man Booker, the Pulitzer, and the American Book Award. We then recorded the titles that these awards had either shortlisted or selected for these awards in the year from December 2017 to December 2018, which produced 40 titles in total. We then took a sample of the books that occupied the number one position in the *New York Times* best-seller list over the same time period; this gave 35 titles.

Using these 75 titles as search terms, we scraped Twitter from December 2017 to December 2018. This was performed using the Twint library, which is a python package that accesses with Twitter via the html public web interface. The advantage of Twint is that it does not impose rate limits, though as an open source project it is less reliable than the official Twitter API. This produced a total of 233,360 tweets that were authored by 128,134 users. Of the scraped tweets, 46,895 belonged to the ‘literary awards’ category and 186,465 belonged to the ‘best-seller’ category. Using the spaCy text cleaning function developed for the previous studies, tweet text was extracted and regularised. This text was then scored for VAD and absolute words. As anticipated, there was low variation in processed tweet length in words relative to the mean (*M* = 11.87; *SD* = 6.59).

### Results

**Fig 7:**
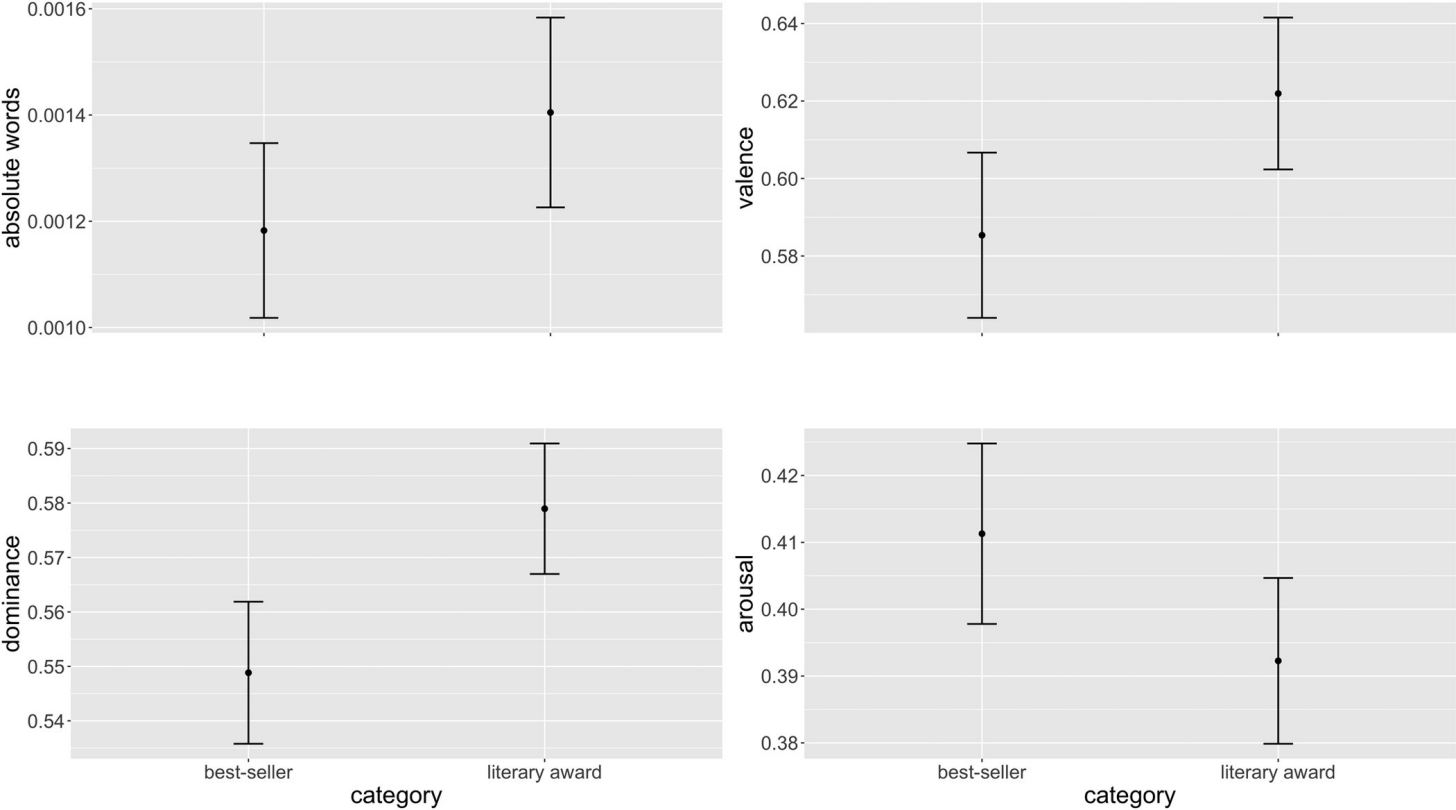
Predicted marginal means for the probability of absolute words, valence, dominance, and arousal.

Valence, arousal, dominance, and absolute words were regressed on multilevel mixed models with random intercepts and random slopes for book title and user. This allowed for the effects of autocorrelation in tweets about the same title and in tweets by the same user. Fixed effects were included for whether or not the category was best-seller or literary award. This initial model failed to converge so the random slopes stipulation was removed in favour of a model with random intercepts only, which reached convergence (see Table 6). Effects were in the predicted directions for valence, arousal, and dominance, but in the opposite direction for absolute words (Fig 7). Since Study 4 also used the same variables as this study, however, a Bonferroni correction was made to allow for multiple comparisons on the same dependent variable; this had the effect of also making arousal statistically non-significant.

**Table 6:** Results of mixed linear models for absolute words, valence, arousal, and dominance regressed on category (best-seller vs literary award) for Study 5.

*DV*	*IV*	β	*SE*	*df*	*t*	*p*	*p*_*corr*_
arousal	(Intercept)	0.411	0.006	70.59	59.7	< .001	*s*
	literary award	-0.019	0.009	70.63	-2.03	0.045	*ns*
valence	(Intercept)	0.585	0.01	70.66	53.79	< .001	*s*
	literary award	0.036	0.014	70.63	2.47	.015	*s*
dominance	(Intercept)	0.548	0.006	70.41	82.46	< .001	*s*
	literary award	0.03	0.009	70.45	3.33	.0013	*s*
absolute words	(intercept)	0.001	8.394 x 10^−5^	47.9	14.08	< .001	*s*
	literary award	0.0002	1.217 x 10^−4^	59.9	1.82	0.072	*ns*

Slopes estimated with respect to book-related subreddit classification.

### Discussion

As always, evidence against the null hypothesis is not necessarily evidence in favour of a competing hypothesis. Nevertheless, the fact that tweets about books featured in literary awards should evince a healthier mood profile with respect to valence and dominance than tweets about best-sellers is a provocative result. In particular, it supports the results for valence and dominance presented in Study 4, as well as validating the supposition that the Study 4 result for arousal is caused by a bimodal latent variable concerned with quality. The result on absolute words was not significant in this case, though the fact that it was in the same direction as in Study 4 indicates that it may be a factor in need of explanation.

In terms of the statistically significant results, we acknowledge that ‘literary award’ and ‘best-seller’ do not map onto ‘book-related’ and ‘non-book-related’, and thus the congruence of results between Study 4 and Study 5 is not a replication. With that said, the categories do cohere with respect to their status as test and control conditions relating to exposure to fiction; in this sense, ‘book-related’ and ‘literary award’ seem to be exercising a similar effect. Across the two studies, the Twitter and Reddit test conditions all have statistically significant differences between each other for valence and dominance, though it is not clear what conclusions should be drawn from this, except perhaps that Reddit and Twitter discourse simply have different emotional profiles.

More generally, the results for the most part support the claim that tweets about literary fiction have a more salutary character than tweets about best-sellers. The reasons why this might be so for valence and dominance have already been explored in the discussion for Study 4 so we will not repeat them here. Though arousal loses significance after the application of a Bonferroni correction, it remains in the predicted direction and no longer exhibits the bimodal distribution found in Study 4. Once again, the absolute words measure is not statistically significant, but this time in the opposite direction to Study 4. On reviewing the absolute words measure in both studies, we note that most comments or tweets had no absolute words in both studies, meaning that a great deal of variation is mapped onto a value of zero for absolute words. This does not invalidate absolute words as a measure of mental distress, but it does contrast unfavourably with the resolution provided by the VAD values, which assign a measure to all observations. It may be, for instance, that high levels of distress are needed to trigger the use of many absolute words, and lower levels are therefore undetected. However, the general point remains that tweets about literary fiction evince a profile more consistent with mental wellbeing than tweets about bestsellers.

### Limitations

Tweeting is not a neutral process; it introduces several confounds that may challenge the results presented here. It is likely, for instance, that social media users who tweet about literary awards are in a relatively secure economic position and well educated—two factors that would impact on well-being. Similarly, as explored in Murray [90], the digital environment selects for certain types of readers and reading experiences that may systematically impact on the kinds of tweets that are produced. The nature of twitter data means it is not possible to control for these issues, but they should still be borne in mind as important considerations.

## General discussion

Across all five studies, a mixed pattern of results emerges. For the two studies in which there was a direct exposure to fiction, no results were recorded; in the three studies that dealt with the recall and discussion of fiction, results were on the whole supportive of the view that engaging with fiction has positive impacts on well-being. For Study 1, this relates to the outcome measures associated with the CORE instrument; in Study 4 and Study 5, the measure was mean change on valence, arousal, and dominance affect measures. How are we to make sense of these results?

At the broadest level, the implication of the five studies is to disconfirm what we might term the ‘pharmaceutical’ model of creative bibliotherapy. This is the view that fiction, in virtue of some intrinsic property, has salutary effects on well-being, and can thus be dose-prescribed in much the same way as, say, an antidepressant or a vitamin supplement. Though no authoritative proponent of creative bibliotherapy holds to so simplistic a view as this, it is nevertheless the intuition behind journalistic claims that ‘reading strengthens your brain’ [91], or that ‘books may have as many health benefits as running or eating broccoli’ [92]. But whether one subscribes to the pharmaceutical view or not, our results suggest that direct exposure to fiction does not seem to confer any measurable benefit in the time adjacent to exposure, at least with respect to common standardised test instruments. (Note that Troscianko [2018b] indicates that individuals with eating disorders recall being *harmed* by reading eating-disorder-themed fiction, but her study does not capture whether this was immediate or required reflective consolidation.) We suggest, therefore, that prescribing fiction to bring about a quick amelioration of symptoms is unlikely to work.

What does seem to have an effect, however, are modes of presentation that require indirect engagement with fiction. Study 1 showed this with respect to the effect of recalling reading fiction, Study 4 indicates that discussing books has more salutary effects than discussing non-book-related topics, and Study 5 gives evidence that discussing literary fiction has more positive effects than best-seller fiction. What is common in all cases is an opportunity to reflect on the material that has been read, whether by way of ordinary mnemonic integration or as a necessary preliminary to engaging with the opinions of others. And even in the case of mnemonic integration, this result was demonstrated only for classic literary texts that are the subject of sustained cultural discussion—potentially allowing that there is a social component at work here, too. The question that emerges is why these modes of encounter with fiction should engender positive effects when more direct encounters do not. We propose three explanations, all of which are consistent with each other.

The first explanation is that reading, when conducted on different time horizons, has different effects. Brysbaert (2019) challenges the view that readers have different mental ’gears’ that cause them to read faster or slower, depending on the reading aims. Nevertheless, his results do show that scanning a text and reading a text occurs in give very different results in words-per-minute metrics. As the experimental presentation of texts in Studies 2 and 3 may well have resulted in readers scanning or skimming them instead of attentively reading them, this may have affected the cognitive impact of the relevant texts. In a similar vein, Fabry and Kokkonen [93] make the case for mind wandering as a form on engagement with the text. That is, the reader’s successful engagement with a text is achieved by bringing to bear predictions generated from enculturated knowledge in a dynamical way in response to textual prompts. The failure of Studies 2 and 3 to produce results may therefore be because the experimental design inhibited this process. If so, new designs that respect the potential effects of long-term reading processes are mandated.

One such design would involve a long-term, three condition study, where participants are assigned to a fiction condition and a non-fiction condition, with the fiction condition being subdivided into literary and non-literary categories. All groups would be provided with the same set of interpretive prompts, which would be congruent with all three texts. Control groups would be generated by creating a second arm of the study with the same design but a substantially shorted duration (e.g. 1 year vs 2 weeks). At fixed points over each arm’s duration, participants would be prompted to interpretively engage with their text and a measure of well-being taken. The first prediction is that, in the long term arm, well-being would improve in the fiction condition relative to the non-fiction condition, and that within the fiction condition, participants in the literary category would improve most. The second prediction is that improvements would only occur in the long-term arm of the study and not the short term one.

Our second explanation of our results centres on social processing; it claims that fiction is an intrinsically social phenomenon, and thus that the positive impacts of fiction on well-being will most visibly manifest in social contexts. That fiction may be intrinsically social arises from evolutionary arguments concerning its utility. One of the more puzzling aspects of fiction from an evolutionary perspective has always been that it should exist at all. Fiction not alone communicates explicitly false information—it does so in a way that uses up temporal, cognitive, and material resources, such as by causing us to care about characters that do not, and often could not, exist. Known as the paradox of fiction [94], this problem has directly or indirectly exercised a number of evolutionary and cognitive theorists [86, 87, 95–100]. One proposed solution is that stories exist as a cultural tool for facilitating large-group living [101–103]. In this view, the performance and discussion of fictional narratives can create prosocial dispositions by activating shared frames of reference and a collective orientation towards the future [81, 104]. In Terence Cave’s words, ‘literature promotes its own downstream conversation, where it becomes mingled with the everyday, the social, the ethical, the political’ [105]. Where this potentially impacts on mood state is through the opioid system. The brain opioid theory maintains that social bonding is experienced as positively valent because it stimulates the endogenous production of *μ*-opioids during affiliative behaviour [106–108]. If so, the positive impact of fiction on well-being and mood derives from the activation of endogenous opioid production through the mechanism of social bonding. Evidence that narratives in general can activate the opioid system is already provided in Dunbar et al. (2016), which shows that group exposure to an emotionally arresting short film can increase pain thresholds relative to less febrile narratives (increased pain threshold is a proxy for opioid system activation). The specific efficacy of literary fiction can, on this basis, be explained as the result of literary fiction providing a better focus for social bonding, possibly by way of its status as a culturally prestigious aesthetic form.

A useful paradigm for testing the social processing explanation is offered in Tarr, Launay, Benson, & Dunbar [109], where administration of naltrexone is used to blockade the endogenous opioid system. That is, Tarr and colleagues conducted a naltrexone-placebo double blind trial to determine whether increases in positive mood, pain threshold and self-reported social closeness between strangers following synchronised dance is suppressed in the test condition. A methodologically straightforward extension of this method would involve exposing matching cohorts to the same fictional materials and subsequently inviting both to participate in separate group discussions of this fiction, with the test group being administered naltrexone before each session. The social processing explanation predicts that discussions in the naltrexone condition should score lower on valence and dominance, as well as on other measures of affect like POMS.

Our second explanation centres on the distinction between lived experience and recalled experience. Specifically, Kahneman & Riis [110] make the useful theoretical distinction between the experiencing self and the evaluating self, with the experiencing self comprehending the moment-to-moment flux of phenomenal experience and the evaluating self taking in the retrospective summation of these experiences. Though the evaluating and experiencing selves often align, there are also many circumstances when they do not—and in these circumstances is typically the judgments of the evaluative self that persist. For instance, the predicted, experienced, and recalled enjoyment of a vacation often differ [111]—but it is the recalled enjoyment that makes choosing to repeat the experience most likely [112]. These systematic differences suggest that the findings generated here may be explicable as the result of the evaluating self extracting therapeutic value from an experience in a way that was not possible for the experiencing self. Literature, it has often been suggested, combines sensorimotor effects with intellectual patterns in a way that secures cognitive engagement that is greater than would result from either factor alone [105, 113–115]. If so, then time-dependent cognitive processes will have had no opportunity to take effect upon immediate exposure; instead, there will be a minimum period of reflective processing that allows for these processes to supervene. (Or it may be that these processes are accelerated by discussion, which makes the reflective processing of others available.) Whatever the precise mechanism, the general point is that literature seems especially engaging of the evaluative rather than the experiencing self, and that this may account for the results presented here.

With respect to testing the experience-evaluation hypothesis, we propose a multiple cohort, staggered timeline experiment. This would involve *n* experimental groups, where each group is divided into test and control conditions. Individuals in the test condition would be exposed to one of a set of fictional literary texts; individuals in the control condition to a neutral textual stimulus. At timepoint *t*_*0*_, all groups will be exposed to their relevant text and a measure of impact on wellbeing taken. At *t*_*1*_, all groups will be prompted to interpretively elaborate on same text, except for one group which will be dropped; at *t*_*2*_, another prompt will be issued, and a second group dropped; this procedure will continue until timepoint *t*_*n*_ is reached, when only one group remains. At timepoint *t*_*n+1*_ all participants will be asked to evaluate the impact on wellbeing of the text they have been exposed to using the same instrument as at *t*_*0*_. The experience-evaluation hypothesis predicts (1) that impact on wellbeing will be greater in the test over the control conditions, and (2) that wellbeing will scale positively with the number of interpretive engagements.

In proposing these studies, our first intention is to gain clarity on the results outlined here. However, we are also conscious of the fact that there is paucity of designs in experimental literary studies. As noted by van Peer and Pander Maat [116], “The problem with literary studies is that the given assumptions are usually vague and general—though often strong and unqualified—and have not been tested with real readers”.

To conclude, we maintain that our studies offer evidence in support of the view that fiction can have a positive impact in well-being. However, these effects do not seem to be realised in peculiarly modern models of reading, where the primary response of the reader is assumed to occur during the privacy of the reading process. Instead, fiction’s positive impact on well-being seems to require processes of mnemonic or social evaluation before it can occur. This result is less important for its positive content—that discussion and reflection amplify the impact of fiction was always known to educators and readers—than it is for what it rules out. If fiction is to be leveraged for therapeutic value, then it cannot be operationalised as a type of cultural pharmacy or—worse—as a cultic object that cures through mere exposure. Instead, it needs to be supported by an infrastructure that systematically and patiently encourages readers to evaluate their experiences by way of reflection and discussion. Inevitably, more research is needed, both with respect to fiction and other modes of literature not discussed here, before more concrete recommendations can be offered. However, we hope to have pointed towards some of the directions this research might take.
